# The relation of ras family oncogene expression to conventional staging criteria and clinical outcome in colorectal carcinoma.

**DOI:** 10.1038/bjc.1986.40

**Published:** 1986-02

**Authors:** I. B. Kerr, D. A. Spandidos, I. G. Finlay, F. D. Lee, C. S. McArdle

## Abstract

The elevated levels of ras-related cellular RNA in a series of colorectal carcinomata (n = 12) was correlated with conventional staging criteria (tumour morphology and Dukes' staging) and with clinical outcome with particular reference to the development of metastatic disease. No direct relationship was evident between these parameters suggesting that although abnormal expression of ras oncogenes may be critical in the development of malignancy, variations in the level of their expression do not appear to be directly related to clinically evident phenotypic differences.


*Br. J. Cancer* (1986), **53**, 231–235

### The relation of *ras* family oncogene expression to conventional staging criteria and clinical outcome in colorectal carcinoma

I.B. Kerr[1,2], D.A. Spandidos[1,3], I.G. Finlay[4], F.D. Lee[2] & C.S. McArdle[4]

[1]*Beatson Institute for Cancer Research, Garscube Estate, Bearsden, Glasgow, UK;* [2]*University Department of Pathology, Royal Infirmary, Glasgow, UK;* [3]*Hellenic Institute Pasteur, Athens, Greece;* [4]*University Department of Surgery, Royal Infirmary, Glasgow, UK.*

**Summary** The elevated levels of *ras*-related cellular RNA in a series of colorectal carcinomata ($n = 12$) was correlated with conventional staging criteria (tumour morphology and Dukes' staging) and with clinical outcome with particular reference to the development of metastatic disease. No direct relationship was evident between these parameters suggesting that although abnormal expression of *ras* oncogenes may be critical in the development of malignancy, variations in the level of their expression do not appear to be directly related to clinically evident phenotypic differences.

The characterisation of transforming cellular oncogenes in a variety of human tumours has contributed an initial understanding of cancer and the process of carcinogenesis at the molecular level (for a review see Cooper, 1984), although the extent to which abnormal expression of these genes is directly related to variations in the clinical behaviour of tumours is not yet clear. Elevated levels of transcription of several oncogenes have now been shown in a wide range of tumours (Slamon *et al.*, 1984; Spandidos & Agnantis, 1984; Spandidos *et al.*, 1985) and it has recently been shown that amplification of an N-*myc* oncogene is related to tumour stage in neuroblastoma (Brodeur *et al.*, 1984) whilst a study of c-*myc* expression in a series of human leukaemias showed increased expression to be apparently related to a more immature phenotype (Birnie *et al.*, 1986). Activation of a *ras* oncogene has also been suggested to be related, in a mouse lymphoma, to the generation of a more aggressive tumour variant (Vousden & Marshall, 1984).

Staging of colorectal carcinoma is currently undertaken using Dukes' classification (Dukes & Bussey, 1958) based on the extent of tumour invasion and spread through bowel wall. However, it has long been recognised that this classification constitutes no more than a relatively crude indication of prognostic probability (Woods, 1980), and the clinical outcome for patients within the different stages may vary significantly. Tumour morphology similarly yields only a very broad correlation with prognosis with marked inconsistencies occurring between degree of differentiation and clinical outcome (Finlay & McArdle, 1982). Despite initial enthusiasm regarding the potential of carcinoembryonic antigen as a tumour marker (see NIH Consensus Statement, 1981) it has now been shown that the presence of elevated preoperative serum levels does not correlate with clinical outcome (Lewi *et al.*, 1984) and that subsequent elevations are not an invariable feature of advanced recurrent or disseminated disease (Finlay & McArdle, 1983). Studies of ploidy in these tumours have shown in general an inverse relation between increasing degrees of aneuploidy and prognosis although this is not absolute (Wolley *et al.*, 1982). We have recently shown, in a study of 'occult' metastatic disease using computerised tomography (Finlay & McArdle, 1982), that the presence or absence of metastatic disease at the time of clinical presentation, long known to be associated with a dire prognosis (Bengtsson *et al.*, 1981), is in fact the most critical prognostic factor, regardless of Dukes' staging and accounts almost entirely for the anticipated pattern of mortality. Studies of the rate of growth of these metastases show that they in fact had been present for a mean period of 3 years prior to surgery. This raises the possibility that these tumours may behave consistently from an early stage as metastasising or non-metastasising variants (Finlay *et al.*, 1982). However the phenotypic determinants or, possibly, features of a host response which might be responsible for such variation remain poorly understood and there is in particular no means of predicting the metastatic potential of a tumour, clearly of importance in determining further therapeutic strategies.

Since we have previously reported a variable elevation of expression of *ras* family oncogenes in a

Correspondence: I.B. Kerr.
Received 8 June 1985; and in revised form, 7 October 1985.

© The Macmillan Press Ltd., 1986

series of premalignant and malignant tumours of the colorectum as compared to normal colonic mucosa (Spandidos & Kerr, 1984), it was obviously of interest to determine whether the variation in expression we observed was related in any way to conventional staging criteria and whether, in particular, this could be used to predict the clinical behaviour of these tumours.

### Materials and methods

Random blocks from a series of tumours and corresponding colonic mucosa were dissected out from operative specimens and snap frozen in liquid nitrogen. Our procedure for extraction of RNA for spot hybridisation assays has already been described in detail. (Spandidos & Kerr, 1984). Briefly 10 $\mu g$ aliquots of total cellular RNA were hybridised sequentially on nitrocellulose paper with $^{32}P$-labelled nick-translated HiHi3 (Ellis *et al.*, 1980) and BS9 (Ellis *et al.*, 1981) recombinant probes containing the viral Kirsten *ras* and viral Harvey *ras* DNA sequences and, subsequently, as controls to check the quantity of RNA present, with the pL335 (Dalla Favera *et al.*, 1982), pAM91 (Minty *et al.*, 1982) and pHR28 probes containing the human cellular *sis*, mouse actin and human 28S ribosomal DNA sequences respectively. Similarly, the quality of our RNA preparations were checked by formaldehyde-agarose gel electrophoresis, followed by ethidium bromide staining, blotting onto nitrocellulose and hybridisation to DNA probes.

Quantitation of the spot hybridisation reactions following autoradiographic exposure was performed by densitometric scanning as previously described (Spandidos *et al.*, 1981) and our figures were based on an arbitrary value obtained when the *ras* probes were hybridised with normal mucosa.

Histopathological reports were based on standard 6 $\mu m$ paraffin embedded sections stained with haematoxylin and eosin.

Patients in the study were followed up as out-patients at 3 monthly intervals with biannual ultrasonography and further radiological investigations as indicated.

### Results

It is evident from Figures 1 and 2 that the degree of elevated expression of Kirsten or Harvey *ras* related sequences in our series does not appear to correlate with either tumour morphology or Dukes' staging since the range of values obtained, ranging from ~2 to 20 fold for the Kirsten to 1.5 to 14 fold for the Harvey-related sequences, clearly overlaps all histological grades and Dukes' stages.

**Table I** Relation of Ki and Ha-*ras* related oncogene expression to age, sex and clinical outcome.

| *Patient* | *Age* | *Sex* | *Follow-up in months* | *Status* | *Ki*-ras | *Ha*-ras |
|---|---|---|---|---|---|---|
| 1 | 52 | M | 27 | ND | 6.3 | 4.5 |
| 2 | 83 | F | (9) | ND[a] | 3.5 | 14 |
| 3 | 62 | F | (19) | DD[a] | 19.0 | 9.0 |
| 4 | 61 | F | 33 | LD | 7.0 | 7.6 |
| 5 | 60 | M | (5) | DD[a] | 3.5 | 1.5 |
| 6 | 54 | F | (6) | DD[a] | 6.5 | 1.5 |
| 7 | 81 | F | (4) | DD[a] | 7.0 | 1.5 |
| 8 | 72 | M | 24 | ND | 9.0 | 1.5 |
| 9 | 75 | M | 36 | ND | 5.0 | 1.5 |
| 10 | 78 | F | (—) | ND[a,c] (post op.) | 4.4 | 1.5 |
| 11 | 79 | M | (—) | —[a,b] (post op.) | 6.5 | 11 |
| 12 | 51 | F | (8) | DD[a] | 1.9 | 14 |

Status of patients at follow-up or death was assessed and abbreviated as follows: ND—no evidence of disease; DD—disseminated disease; LD—local disease; [a]dead; [b]not known; [c]post-mortem assessment.

Although follow-up is in some cases relatively short it is, however, known that of those who will ultimately develop disseminated disease the majority would be expected to do so within a period of two years (Finlay *et al.*, 1982*c*). The information in Table I thus shows that the level of expression of these sequences does not appear to be related to clinical outcome in general nor to the development in particular of metastatic disease.

### Discussion

Our results therefore indicate that, although some elevation of Kirsten and Harvey-related *ras* oncogene expression is seen in all these tumours, variation in the amounts of RNA homologous to the *ras* probes is not related to clinically apparent phenotypic variation nor clinical outcome. If the hypothesis relating to a heterogeneity of cells with metastatic potential in a primary tumour (Hart & Fidler, 1981), currently held by several authors to be extremely contentious (Weiss *et al.*, 1983; Alexander, 1983), were indeed true in this tumour type, then random sampling could obviously not be expected to identify such variation. We have, however, already presented evidence showing that these tumours appear to behave consistently as metastasising or non-metastasising variants (Finlay *et al.*, 1982). In addition, although a heterogeneous

**Figure 1** Relation of (a) Ki-*ras* and (b) Ha-*ras* related oncogene expression to Dukes' stage (i) and degree of tumour differentiation (ii). In one case histology was not available.

pattern of p21 expression has been reported in formalin-fixed paraffin-embedded sections of these tumours by immunocytochemical techniques (Thor *et al.*, 1984), we (Kerr *et al.*, 1985) and others (Williams *et al.*, 1985) have demonstrated, using the monoclonal antibody Y13-259 (Furth *et al.*, 1982) on frozen sections of these tumours, widespread positive staining of tumour cells with little reactivity of underlying stroma. It is unfortunately not possible to quantitate expression using such techniques. Thus, although some variation within individual cells cannot be excluded, it is clear from the evidence based on presently available methods of study that neither the presence of *ras* oncogene expression nor observable differences in its level can be used to predict variations in clinical behaviour in these tumours.

These findings are consistent with a recently published study which showed similar elevations of p21 protein levels in these tumours but which was also unable to correlate these absolutely with conventional staging criteria, although, interestingly, it was shown that metastatic tumour contained relatively low levels of p21 (Gallick *et al.*, 1985).

Although activity of a number of cellular proto-oncogenes has been demonstrated in a variety of tissue types and at various developmental stages (Westin *et al.*, 1982*a,b*; Muller *et al.*, 1982, 1983), it is not yet clear in the majority of cases what their exact physiological roles might be. It is widely hypothesised, however, that they may be concerned with cell growth control and regulation (Heldin & Westermark, 1984). Nor is it precisely understood what part they play in the process of carcinogenesis although they have been identified in several cases as the active transforming genes in 3T3 transfection assays, including an activated Ki-*ras* from a colonic carcinoma (Pulciani *et al.*, 1982). We have previously shown elevated expression of *ras* family oncogenes in malignant and premalignant tumours of the colorectum and elevated expression as well as transforming activity of a Ha-*ras* oncogene has been shown in experimentally induced mouse skin carcinomata and in premalignant papillomata (Balmain *et al.*, 1984). It is also of interest in this context that during experimental *in vitro* transformation of early passage rodent cells, transfection with either an activated, mutated Ha-*ras* oncogene or with a normal Ha-*ras* proto-oncogene linked to an enhancer induces immortalisation alone, and that only when linked to transcriptional enhancers can a mutated form of the gene induce complete malignant transformation (Spandidos & Wilkie, 1984). Such results would be consistent with a role for both quantitative and qualitative changes in *ras* gene expression within an overall multi-step process of carcinogenesis.

If indeed, as we have previously hypothesised, activation of these genes at a premalignant stage may be critical in the process of carcinogenesis but not in itself sufficient, it may be that subsequent event(s) whose nature is not yet clear, perhaps involving activation of a variety of other gene loci and possibly associated with some form of genomic instability, are more directly involved in the expression of a frankly malignant phenotype and its variants. It is, however, possible that variation in the nature of the mutation previously shown to be associated with *ras* activation (Reddy *et al.*, 1982; Shimizu *et al.*, 1983; Santos *et al.*, 1984) may also be significant in this context, although in a series of urothelial tumours recently reported (Fujita *et al.*, 1984) neither the presence of a transforming *ras* oncogene in DNA in a small percentage of cases nor the presence or absence of a documented mutation in these, appeared to be related to tumour stage. Nonetheless, study of such variation and of abnormal expression of cellular oncogenes, already shown in the context of neuroblastoma even in gross tumour specimens to be clinically significant, as well as of other event(s) involved in the generation of malignancy will clearly be important in attempting to define the behaviour of these tumours more fully.

We thank members of the Departments of Pathology and Surgery, Glasgow Royal Infirmary, for their cooperation in obtaining and documenting specimens and Dr G.D. Birnie for critical appraisal of the manuscript. This work was supported by the Cancer Research Campaign of Great Britain.

### References

ALEXANDER, P. (1983). Dormant metastases – studies in experimental animals. *J. Pathology*, **141**, 379.

BALMAIN, A., RAMSDEN, M., BOWDEN, G.T. & SMITH, J. (1984). Activation of the mouse cellular Harvey-*ras* gene in chemically induced benign skin papillomas. *Nature*, **307**, 658.

BENGTSSON, G.B., CARLSSON, G., HAFSTROM, L. & JONSSON, P. (1981). Natural history of patients with untreated liver metastases from colorectal cancer. *Am. J. Surg.*, **141**, 586.

BIRNIE, G.D., WARNOCK, A.M., BURNS, J.H. & CLARK, P. (1986). Expression of the *myc* gene locus in populations of normal leucocytes from leukaemia patients and normal individuals. *Leuk. Res.* (in press).

BRODEUR, G.M., SEEGER, R.C., SCHWAB, M., VARMUS, H.E. & BISHOP, J.M. (1984). Amplification of N-*myc* in untreated human neuroblastomas correlates with advanced stage. *Science*, **224**, 1121.

COOPER, G.M. (1984). Activation of transforming genes in neoplasms. *Br. J. Cancer*, **50**, 137.

DUKES, C.E. & BUSSEY, H.J.R. (1958). The spread of rectal cancer and its effects on prognosis. *Br. J. Cancer*, **12**, 309.

ELLIS, R.W., DEFEO, D., SHIH, T.Y. & 5 others. (1981). The p21 *src* genes of Harvey and Kirsten sarcoma viruses originate from different members of a family of normal vertebrate genes. *Nature*, **292**, 506.

ELLIS, R.W., DEFEO, D., MARYHAK & 5 others. (1980). Dual evolutionary origin for the rat genetic sequences of Harvey murine sarcoma virus. *J. Virol.*, **36**, 408.

FINLAY, I.G. & McARDLE, C.S. (1982). The identification of patients at high risk following curative resection for colorectal carcinoma. *Br. J. Surg.*, **69**, 583.

FINLAY, I.G., BRUNTON, E.F., MEEK, D. & McARDLE, C.S. (1982). Rate of growth of hepatic metastases in colorectal carcinoma. *Br. J. Surg.*, **69**, 689.

FINLAY, I.G. & McARDLE, C.S. (1983). Role of carcinoembryonic antigen in detection of asymptomatic disseminated disease in colorectal carcinoma. *Br. Med. J.*, **286**, 1242.

FUJITA, J., YOSHIDA, O., YUASA, Y., RHIM, J.S., HANAKA, M. & AARONSON, S.A. (1984). Ha-*ras* oncogenes are activated by somatic alterations in human urinary tract tumours. *Nature*, **309**, 464.

FURTH, M.E., DAVIS, L.J., FLEURDELYS, B. & SCOLNICK, E.M. (1982). Monoclonal antibodies to the p21 products of the transforming gene of Harvey murine sarcoma virus and of the cellular *ras* gene family. *J. Virol.*, **43**, 294–304.

GALLICK, G.E., KURZROCK, R., KLOETZER, W.S., ARLINGHAUS, R.B. & GUTTERMANN, J.U. (1985). Expression of p21 *ras* in fresh primary and metastatic human colorectal tumors. *Proc. Natl. Acad. Sci.*, **82**, 1795.

HART, I.R. & FIDLER, I.J. (1981). The implications of tumour heterogeneity for studies on the biology and therapy of cancer metastasis. *Biochem. Biophys Acta*, **651**, 37.

HELDIN, C.H. & WESTERMARK, B. (1984). Growth factors: Mechanism of action and relation to oncogenes. *Cell*, **37**, 9.

KERR, I.B., LEE, F.D., QUINTANILLA, M. & BALMAIN, A. (1985). Immunocytochemical demonstration of p21 *ras* family oncogene product in normal mucosa and in premalignant and malignant tumours of the colorectum. *Br. J. Cancer,* **52,** 695.

LEWI, H., BLUMGART, C.H., CARTER, D.C. & 5 others. (1984). Pre-operative carcino-embryonic antigen and survival in patients with colorectal cancer. *Br. J. Surg.,* **71,** 206.

MINTY, A.J., ALONSO, S., CARAVATTI, M. & BUCKINGHAM, M.E. (1982). A fetal skeletal muscle actin mRNA in the mouse and its identity with cardiac actin mRNA. *Cell,* **30,** 185.

MULLER, R. SLAMON, D.J., TREMBLAY, J.M., CLINE, M.J. & VERMA, I.M. (1982). Differential expression of cellular oncogenes during pre- and postnatal development of the mouse. *Nature,* **299,** 640.

MULLER, R., VERMA, I.M. & ADAMSON, E.D. (1983). Expression of c-*onc* genes: c-*fos* transcripts accumulate to high levels during development of mouse placenta yolk sac and amnion. *EMBO J.,* **2,** 679.

N.I.H. CONSENSUS STATEMENT (1981). Carcino-embryonic antigen: Its role as a marker in the management of cancer. *Br. Med. J.,* **282,** 373.

PULCIANI, S., SANTOS, E., LAUVER, A.V., LONG, L.K., AARONSON, S.A. & BARBACID, M. (1982). Oncogenes in human solid tumours. *Nature,* **300,** 539.

REDDY, E.P., REYNOLDS, R.K., SANTOS, E. & BARBACID, M. (1982). A point mutation is responsible for the acquisition of transforming properties by the T24 human bladder carcinoma oncogene. *Nature,* **300,** 149.

SANTOS, E., MARTIN-ZANCA, D., REDDY, E.P., PIEROTTI, M.A., DELLA PORTA, G. & BARBACID, M. (1984). Malignant activation of a K-*ras* oncogene in lung carcinoma but not in normal tissue of the same patient. *Science,* **223,** 661.

SHIMIZU, K., BIRNBAUM, D., RULEY, M.A. & 6 others. (1983). Structure of the Ki-*ras* gene of the human lung carcinoma cell line Calu-1. *Nature,* **304,** 497.

SLAMON, D.J., DEKERNION, J.B., VERMA, I.M. & CLINE, M.J. (1984). Expression of cellular oncogenes in human malignancies. *Science,* **224,** 256.

SPANDIDOS, D.A. & AGNANTIS, N.J. (1984). Human malignant tumours of the breast as compared to their respective normal tissue have elevated expression of the Harvey *ras* oncogene. *Anticancer Res.,* **4,** 269.

SPANDIDOS, D.A., HARRISON, P.R. & PAUL, J. (1981). Transfer and expression of herpes simplex virus thymidine kinase and human globin genes in mammalian cells studied by spot hybridization. *Biosci. Rep.,* 1911.

SPANDIDOS, D.A. & KERR, I.B. (1984). Elevated expression of the human *ras* oncogene family in premalignant and malignant tumours of the colorectum. *Br. J. Cancer,* **49,** 681.

SPANDIDOS, D.A., LAMOTHE, A. & FIELD, J.K. (1985). Multiple transcriptional activation of cellular oncogenes in human head and neck solid tumours. *Anticancer Res.,* **5,** 221.

SPANDIDOS, D.A. & WILKIE, N.M. (1984). Malignant transformation of early passage rodent cells by a single initiated human oncogene. *Nature,* **310,** 469.

THOR, A., HORAN-HAND, P., WUNDERLICH, D., CARUSO, A., MURARO, R. & SCHLOM, J. (1984). Monoclonal antibodies define differential *ras* expression in malignant and benign colonic disease. *Nature,* **311,** 562.

VOUSDEN, K.H. & MARSHALL, C.J. (1984). Three different activated *ras* genes in mouse tumours: evidence for oncogene activation during progression of a mouse lymphoma. *EMBO J.,* **3,** 913.

WEISS, L., HOLMES, J.C. & WARD, P.M. (1983). Do metastases arise from pre-existing populations of cancer cells? *Br. J. Cancer,* **47,** 81.

WESTIN, E.H., GALLO, R.C., ARYA, S.K. & 5 others. (1982*a*). Differential expression of the amv gene in human haematopoietic cells. *Proc. Natl Acad. Sci.,* **79,** 2194.

WESTIN, E.H., WONG-STAAL, F., GELMANN, E.P. & 5 others. (1982*b*). Expression of cellular homologues of retroviral *onc* genes in human haematopoietic cells. *Proc. Natl Acad. Sci.,* **79,** 2490.

WILLIAMS, A.R.W., PIRIS, J., SPANDIDOS, D.A. & WYLLIE, A.H. (1985). Immunohistochemical detection of the *ras* oncogene p21 product in an experimental tumour and in human colorectal neoplasms. *Br. J. Cancer,* **52,** 687.

WOLLEY, R.C., SCHREIBER, K., KOSS, L.G., KAKAS, M. & SHERMAN, A. (1982). DNA distribution in human colon carcinomas and its relationship to clinical behaviour. *J. Nat. Canc. Inst.,* **69,** 15.

WOODS, C.B. (1980). Prognostic factors in colorectal cancer. In *Recent Advances in Surgery,* Taylor, S. (ed.). Churchill Livingstone: London.


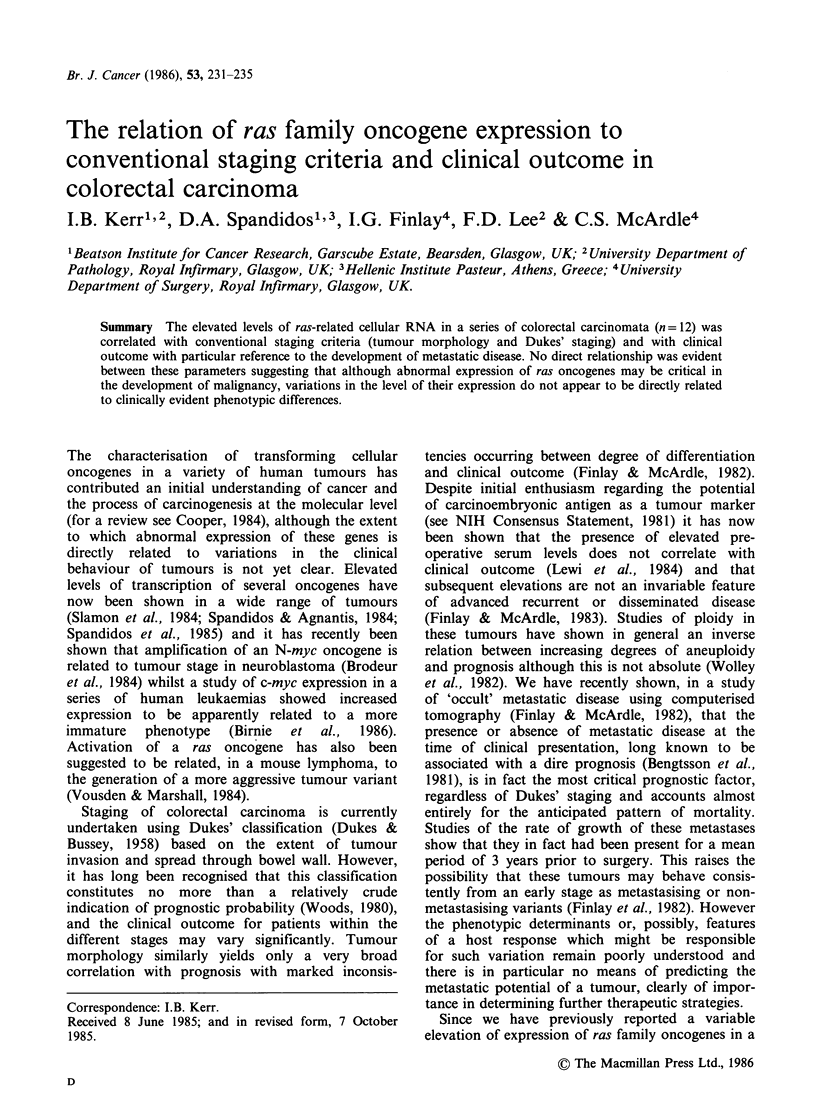

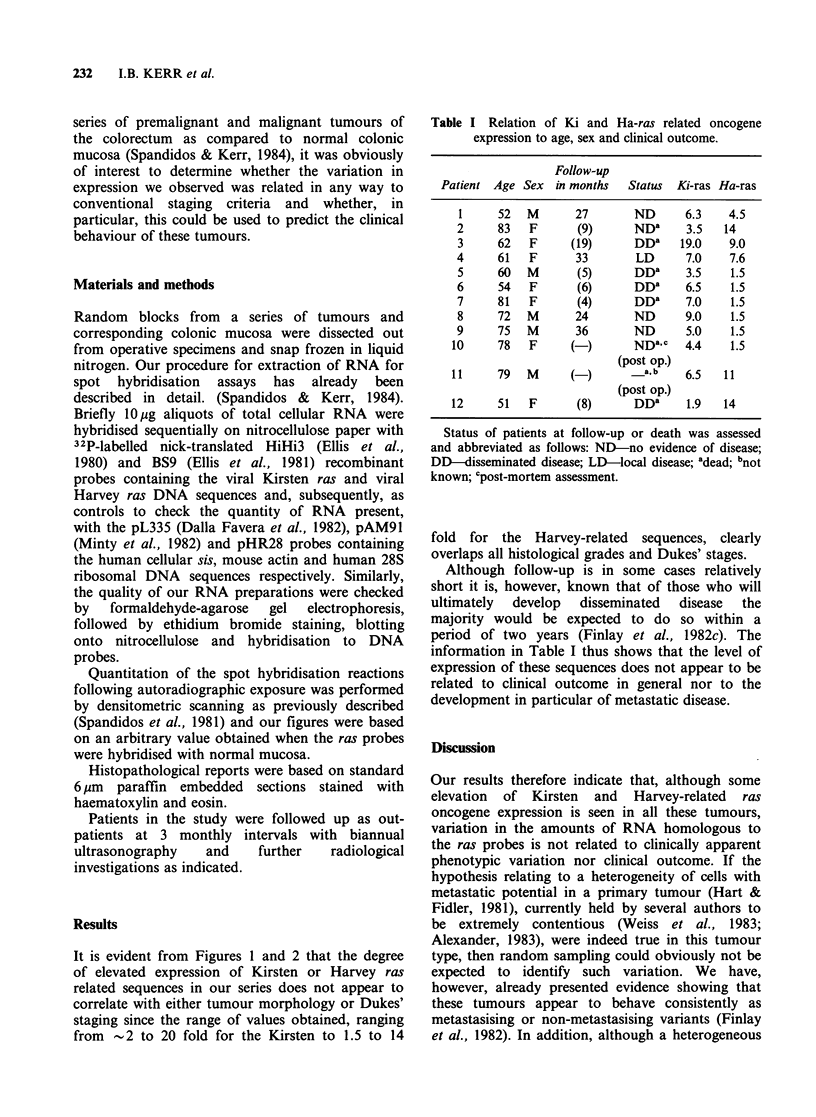

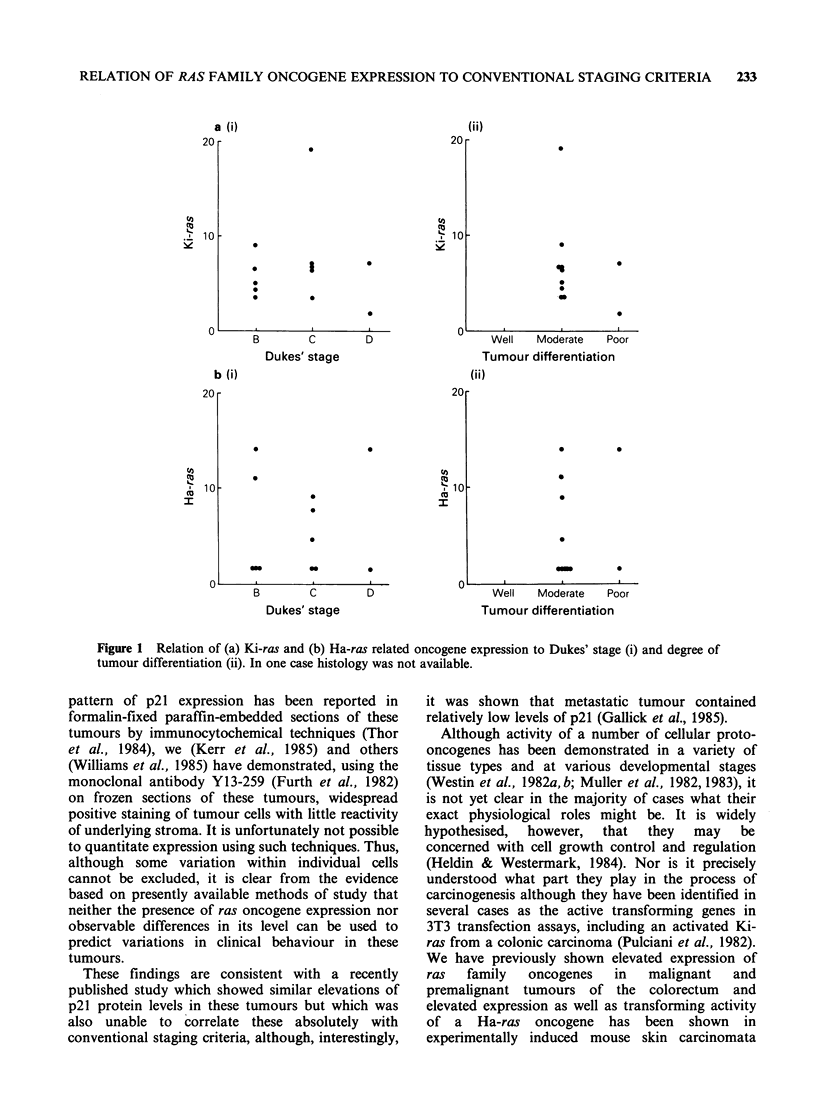

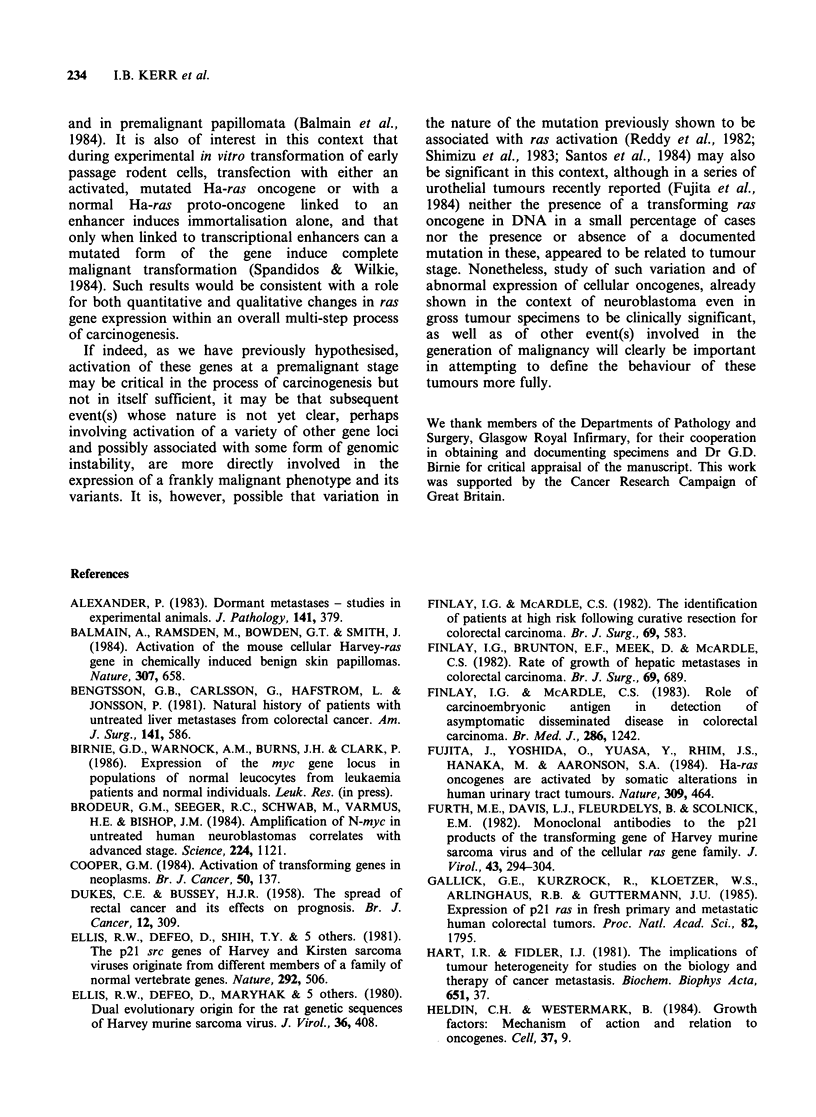

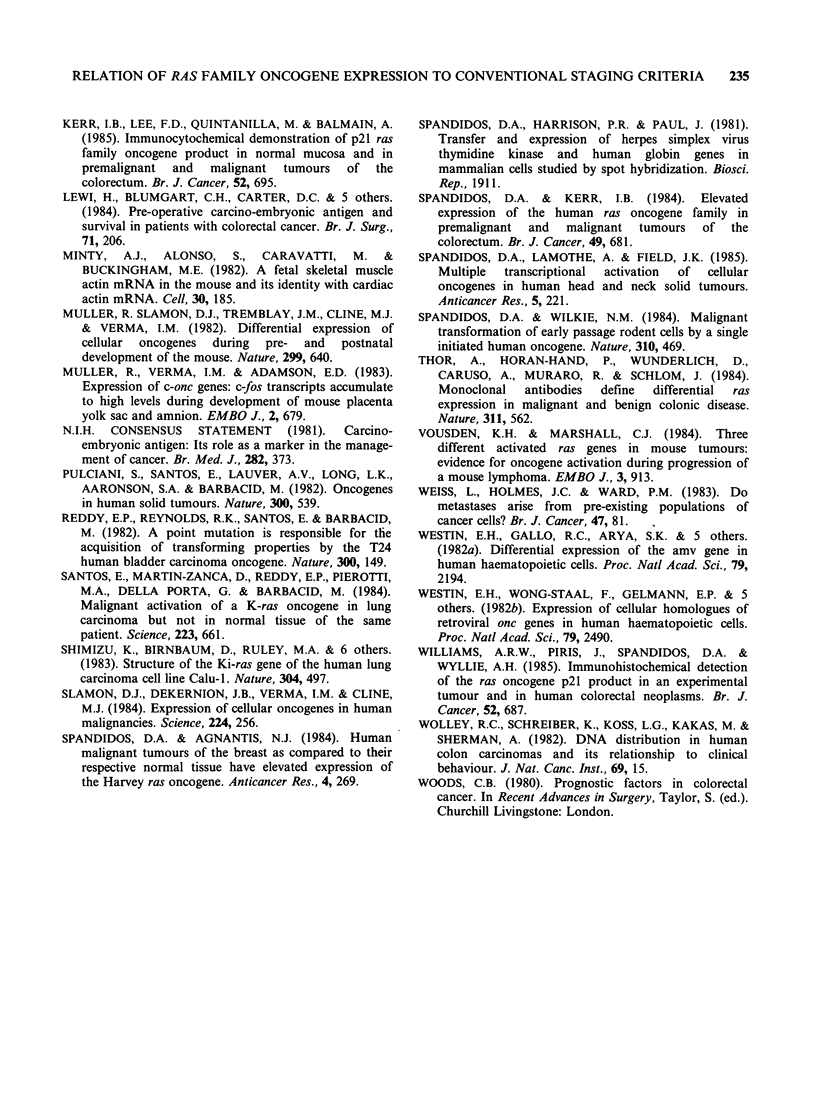


## References

[OCR_00435] Alexander P. (1983). Dormant metastases--studies in experimental animals.. J Pathol.

[OCR_00439] Balmain A., Ramsden M., Bowden G. T., Smith J. (1984). Activation of the mouse cellular Harvey-ras gene in chemically induced benign skin papillomas.. Nature.

[OCR_00445] Bengtsson G., Carlsson G., Hafström L., Jönsson P. E. (1981). Natural history of patients with untreated liver metastases from colorectal cancer.. Am J Surg.

[OCR_00457] Brodeur G. M., Seeger R. C., Schwab M., Varmus H. E., Bishop J. M. (1984). Amplification of N-myc in untreated human neuroblastomas correlates with advanced disease stage.. Science.

[OCR_00463] Cooper G. M. (1984). The 1984 Walter Hubert lecture. Activation of transforming genes in neoplasms.. Br J Cancer.

[OCR_00467] DUKES C. E., BUSSEY H. J. (1958). The spread of rectal cancer and its effect on prognosis.. Br J Cancer.

[OCR_00478] Ellis R. W., DeFeo D., Maryak J. M., Young H. A., Shih T. Y., Chang E. H., Lowy D. R., Scolnick E. M. (1980). Dual evolutionary origin for the rat genetic sequences of Harvey murine sarcoma virus.. J Virol.

[OCR_00472] Ellis R. W., Defeo D., Shih T. Y., Gonda M. A., Young H. A., Tsuchida N., Lowy D. R., Scolnick E. M. (1981). The p21 src genes of Harvey and Kirsten sarcoma viruses originate from divergent members of a family of normal vertebrate genes.. Nature.

[OCR_00493] Finlay I. G., McArdle C. S. (1983). Role of carcinoembryonic antigen in detection of asymptomatic disseminated disease in colorectal carcinoma.. Br Med J (Clin Res Ed).

[OCR_00483] Finlay I. G., McArdle C. S. (1982). The identification of patients at high risk following curative resection for colorectal carcinoma.. Br J Surg.

[OCR_00499] Fujita J., Yoshida O., Yuasa Y., Rhim J. S., Hatanaka M., Aaronson S. A. Ha-ras oncogenes are activated by somatic alterations in human urinary tract tumours.. Nature.

[OCR_00505] Furth M. E., Davis L. J., Fleurdelys B., Scolnick E. M. (1982). Monoclonal antibodies to the p21 products of the transforming gene of Harvey murine sarcoma virus and of the cellular ras gene family.. J Virol.

[OCR_00512] Gallick G. E., Kurzrock R., Kloetzer W. S., Arlinghaus R. B., Gutterman J. U. (1985). Expression of p21ras in fresh primary and metastatic human colorectal tumors.. Proc Natl Acad Sci U S A.

[OCR_00519] Hart I. R., Fidler I. J. (1981). The implications of tumor heterogeneity for studies on the biology of cancer metastasis.. Biochim Biophys Acta.

[OCR_00525] Heldin C. H., Westermark B. (1984). Growth factors: mechanism of action and relation to oncogenes.. Cell.

[OCR_00532] Kerr I. B., Lee F. D., Quintanilla M., Balmain A. (1985). Immunocytochemical demonstration of p21 ras family oncogene product in normal mucosa and in premalignant and malignant tumours of the colorectum.. Br J Cancer.

[OCR_00539] Lewi H., Blumgart L. H., Carter D. C., Gillis C. R., Hole D., Ratcliffe J. G., Wood C. B., McArdle C. S. (1984). Pre-operative carcino-embryonic antigen and survival in patients with colorectal cancer.. Br J Surg.

[OCR_00545] Minty A. J., Alonso S., Caravatti M., Buckingham M. E. (1982). A fetal skeletal muscle actin mRNA in the mouse and its identity with cardiac actin mRNA.. Cell.

[OCR_00551] Müller R., Slamon D. J., Tremblay J. M., Cline M. J., Verma I. M. (1982). Differential expression of cellular oncogenes during pre- and postnatal development of the mouse.. Nature.

[OCR_00557] Müller R., Verma I. M., Adamson E. D. (1983). Expression of c-onc genes: c-fos transcripts accumulate to high levels during development of mouse placenta, yolk sac and amnion.. EMBO J.

[OCR_00568] Pulciani S., Santos E., Lauver A. V., Long L. K., Aaronson S. A., Barbacid M. (1982). Oncogenes in solid human tumours.. Nature.

[OCR_00573] Reddy E. P., Reynolds R. K., Santos E., Barbacid M. (1982). A point mutation is responsible for the acquisition of transforming properties by the T24 human bladder carcinoma oncogene.. Nature.

[OCR_00579] Santos E., Martin-Zanca D., Reddy E. P., Pierotti M. A., Della Porta G., Barbacid M. (1984). Malignant activation of a K-ras oncogene in lung carcinoma but not in normal tissue of the same patient.. Science.

[OCR_00586] Shimizu K., Birnbaum D., Ruley M. A., Fasano O., Suard Y., Edlund L., Taparowsky E., Goldfarb M., Wigler M. (1983). Structure of the Ki-ras gene of the human lung carcinoma cell line Calu-1.. Nature.

[OCR_00591] Slamon D. J., deKernion J. B., Verma I. M., Cline M. J. (1984). Expression of cellular oncogenes in human malignancies.. Science.

[OCR_00596] Spandidos D. A., Agnantis N. J. (1984). Human malignant tumours of the breast, as compared to their respective normal tissue, have elevated expression of the Harvey ras oncogene.. Anticancer Res.

[OCR_00609] Spandidos D. A., Kerr I. B. (1984). Elevated expression of the human ras oncogene family in premalignant and malignant tumours of the colorectum.. Br J Cancer.

[OCR_00615] Spandidos D. A., Lamothe A., Field J. K. (1985). Multiple transcriptional activation of cellular oncogenes in human head and neck solid tumours.. Anticancer Res.

[OCR_00621] Spandidos D. A., Wilkie N. M. (1984). Malignant transformation of early passage rodent cells by a single mutated human oncogene.. Nature.

[OCR_00626] Thor A., Horan Hand P., Wunderlich D., Caruso A., Muraro R., Schlom J. (1984). Monoclonal antibodies define differential ras gene expression in malignant and benign colonic diseases.. Nature.

[OCR_00633] Vousden K. H., Marshall C. J. (1984). Three different activated ras genes in mouse tumours; evidence for oncogene activation during progression of a mouse lymphoma.. EMBO J.

[OCR_00639] Weiss L., Holmes J. C., Ward P. M. (1983). Do metastases arise from pre-existing subpopulations of cancer cells?. Br J Cancer.

[OCR_00644] Westin E. H., Gallo R. C., Arya S. K., Eva A., Souza L. M., Baluda M. A., Aaronson S. A., Wong-Staal F. (1982). Differential expression of the amv gene in human hematopoietic cells.. Proc Natl Acad Sci U S A.

[OCR_00650] Westin E. H., Wong-Staal F., Gelmann E. P., Dalla-Favera R., Papas T. S., Lautenberger J. A., Eva A., Reddy E. P., Tronick S. R., Aaronson S. A. (1982). Expression of cellular homologues of retroviral onc genes in human hematopoietic cells.. Proc Natl Acad Sci U S A.

[OCR_00656] Williams A. R., Piris J., Spandidos D. A., Wyllie A. H. (1985). Immunohistochemical detection of the ras oncogene p21 product in an experimental tumour and in human colorectal neoplasms.. Br J Cancer.

[OCR_00663] Wolley R. C., Schreiber K., Koss L. G., Karas M., Sherman A. (1982). DNA distribution in human colon carcinomas and its relationship to clinical behavior.. J Natl Cancer Inst.

